# Chronic Pain and Biopsychosocial Correlates in Rural Filipino Adults: A Cross-Sectional Secondary Analysis

**DOI:** 10.3390/healthcare14020214

**Published:** 2026-01-15

**Authors:** James Mangohig, Jennifer Kawi, Andrew Thomas Reyes, Reimund Serafica, Marysol C. Cacciata, Carol Manilay-Robles, Lorraine S. Evangelista

**Affiliations:** 1 Physical Medicine and Rehabilitation, Sunrise Health Graduate Medical Education Consortium, Las Vegas, NV 89128, USA; 2 Department of Research, Cizik School of Nursing, The University of Texas Health Sciences Center at Houston, Houston, TX 77030, USA; jennifer.kawi@uth.tmc.edu; 3 School of Nursing, University of Nevada, Las Vegas, Las Vegas, NV 89154, USA; 4 Veteran Affairs Long Beach Healthcare System, Long Beach, CA 90822, USA; marysol_cacciata@yahoo.com; 5 Arleigh Burke Pavilion, McLean, VA 22101, USA; 6 Sue & Bill Gross School of Nursing, University of California, Irvine, Irvine, CA 92697, USA

**Keywords:** chronic pain, social determinants of health, rural health, Philippines, cross-sectional study

## Abstract

**Background**: Chronic pain is a leading contributor to disability worldwide, yet population-based data from rural and medically underserved settings remain limited. Evidence describing the biopsychosocial correlates of chronic pain in rural Filipino communities is particularly scarce. **Methods**: We conducted a secondary analysis of cross-sectional data from the I-HELP-FILIPINO community cohort, collected between January and June 2017. Participants included 909 adults aged 18–93 years who voluntarily attended barangay clinic days in rural Philippine communities. Pain severity was assessed with standardized self-report tools. Sociodemographic, psychosocial, sleep, and functional health factors were examined using bivariate analysis and hierarchical linear regression. Results are shown with 95% confidence intervals. **Results**: Chronic pain was very common, affecting 83.8% of participants, with 5.6% experiencing severe pain. In multivariable models, psychosocial distress, sleep issues, and reduced physical functioning were significantly linked to increased pain severity, while demographic factors contributed minimally to the variance. Most of the explained variance in pain outcomes was accounted for by psychosocial and clinical variables. **Conclusions**: In this rural Filipino cohort, chronic pain was strongly associated with modifiable psychosocial, sleep, and functional health factors. Focusing on community-based and culturally tailored interventions can inspire hope and empower healthcare professionals and policymakers to tackle these issues more effectively.

## 1. Introduction

Chronic pain, defined as pain persisting for at least three months, affects a substantial proportion of adults worldwide and is a major factor in disability and reduced quality of life [1,2]. Recent surveillance data highlight the magnitude and persistence of this issue; for instance, national estimates from the U.S. indicate that approximately one in four adults suffers from chronic pain, with a considerable subset experiencing high-impact pain that limits daily activities or employment [3]. These national estimates are consistent with global rates of up to 24% showing greater prevalence among individuals living in rural areas compared urban populations [4]. Chronic pain often coexists with sleep disturbances, emotional disorders, functional impairment, and social isolation, exacerbating suffering and complicating effective care [1,5].

The biopsychosocial model views pain as the result of interactions among biological, psychological, and social factors [1,6]. Social determinants of health, including education, income, insurance coverage, and access to services, influence the risk of chronic pain and the feasibility of multimodal, nonpharmacologic interventions. Challenges are notably significant in rural areas due to geographic isolation, restricted availability of specialists, and increased structural barriers that limit access to evidence-based treatments and coordinated pain services [1,7]. Chronic pain care benefits from coordinated, interdisciplinary pathways that align clinical goals with health system priorities, such as patient outcomes and experience, cost stewardship, and workforce well-being—core goals reflected in the Quadruple Aim framework [5,8].

Clinical networks and integrated care frameworks provide a practical approach to enhance coordination and standardize routes in environments with limited specialty pain resources. Evidence indicates that clinical networks can enhance quality improvement via common recommendations, optimized referral pathways, and uniform application of clinical tools—strategies that can elevate service delivery and, in certain settings, patient outcomes [9,10]. Recent research highlights the ability of clinical networks to improve organizational efficiency and support practice implementation, which is crucial for the growth of low-intensity, community-based pain care [11]. Despite the growing global emphasis on integrated models, most epidemiological evidence informing pain policy and clinical guidance primarily comes from high-income countries. This limitation restricts its relevance to low- and middle-income contexts and underserved rural populations [4,7].

Specifically, rural communities and geographically isolated regions in the Philippines are inadequately represented in population-level pain studies. Limited research has investigated the combined influence of clinical factors (e.g., sleep disturbances and physical functioning), psychological factors (e.g., depression and anxiety), and socioeconomic determinants on pain severity, employing hierarchical methods that assess the additional impact of psychosocial and clinical domains beyond fundamental demographics. Empirical data from rural Filipino settings are therefore needed to (a) inform feasible assessment priorities in primary care and community outreach, (b) identify modifiable targets for low-intensity interventions (e.g., sleep and mood), and (c) support policy planning for equitable, multimodal pain care.

To address these gaps, the present study is a secondary analysis of cross-sectional data from a community-based cohort of adults residing in rural Filipino communities, collected in 2017 through barangay clinic days and community outreach activities. Although the dataset is not recent, comparable population-based rural datasets in the Philippines that include detailed psychosocial, sleep, and functional measures remain scarce while rural conditions remain unchanged, underscoring the continued value of these data for clinical planning and health system design especially in underserved communities. Consequently, the primary objectives of this study were to estimate the prevalence and severity of chronic pain and to quantify the independent associations of psychosocial and clinical factors with pain severity. The secondary objectives were to evaluate the contribution of sociodemographic and access-related factors to pain severity and to compare the variance explained by demographics alone with that of models incorporating psychosocial and clinical domains using hierarchical regression. Guided by the biopsychosocial framework, we hypothesized that sleep disturbance, poorer physical functioning, and higher levels of depression and anxiety would explain substantially more variance in pain severity than demographic factors alone.

## 2. Materials and Methods

### 2.1. Study Design and Reporting Standards

This study provides a secondary analysis of cross-sectional data from the I-HELP-FILIPINO community cohort. The cohort was established to examine health conditions and psychosocial factors among adults living in rural, underserved barangays in the Philippines [11]. The data utilized for this analysis was systematically collected from January to June 2017 during designated barangay clinics and community outreach initiatives.

This research complies with the Strengthening the Reporting of Observational Studies in Epidemiology (STROBE) guidelines for cross-sectional studies, thereby promoting transparency, reproducibility, and clarity regarding methods and findings [12]. The design, setting, participant selection, variables, data sources, and analytic procedures were thoroughly documented in accordance with the STROBE checklist.

As a secondary analysis, no additional data were collected for this study. The analytic plan, including variable selection and model specification, was informed by the biopsychosocial framework and developed a priori to examine the relative contribution of sociodemographic, psychosocial, sleep, and functional health factors to chronic pain severity. All analyses were observational in nature, and causal inferences were not intended.

### 2.2. Setting, Sampling and Recruitment

The research was conducted in rural barangays in the Philippines that are medically underserved due to geographic remoteness, restricted access to health services, and insufficient healthcare resources. Data were gathered during designated barangay clinic days and community outreach events conducted as components of the I-HELP-FILIPINO community health campaign. The research was facilitated by local barangay health workers and municipal health departments through the coordination of community outreach activities and the provision of logistical support during clinic days.

**Figure 1:**
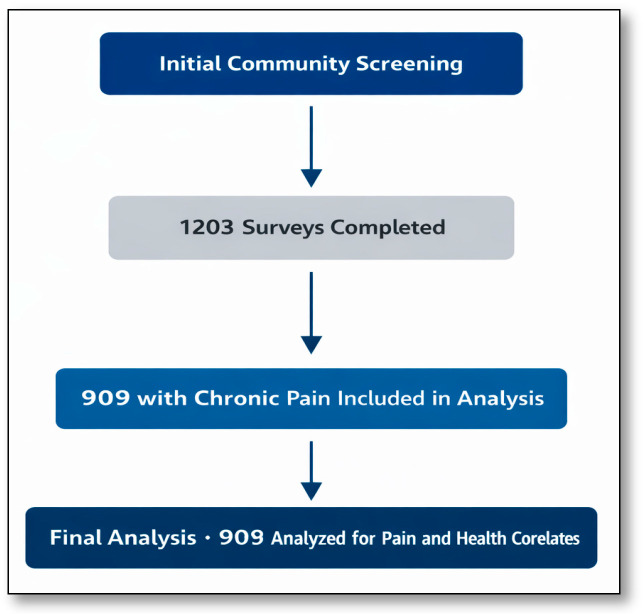
Study flow diagram of participant selection.

A convenience sampling method was employed. Adults aged 18 years and older who attended clinic days, or outreach events were eligible to participate. Participation was entirely voluntary and self-initiated. Individuals were informed that a health survey was available, and those who chose to participate completed the questionnaire on site. Study staff did not approach, solicit, or recruit individuals directly. As a result, only individuals who chose to participate were enrolled, and there were no formally identified non-participants or recruitment denominators (Figure 1).

Because recruitment was limited to individuals who attended barangay clinics or outreach events, the sample may not be representative of all adults residing in the participating communities, particularly those who do not routinely access health services. This constraint is acknowledged and further detailed in the Limitations section. Nonetheless, clinic-based sampling remains a pragmatic and prevalent technique for community health research in rural and resource-constrained regions, where population registries and door-to-door methods may be impractical.

### 2.3. Participants and Eligibility Criteria

Participants were adults aged 18 years or older who attended barangay clinic days or community outreach events during the data collection period and voluntarily filled out the health survey. Eligibility criteria for the parent cohort included residency in participating rural barangays and the ability to provide informed consent. There were no upper age limits, which led to a wide age range that mirrors the demographic makeup of rural communities.

For the present secondary analysis, participants were included if they had complete data on pain severity and key covariates of interest, including sociodemographic characteristics, psychosocial measures, sleep disturbance, and physical functioning. Individuals with missing data on the primary outcome variable were excluded from the analytic sample.

Age was analyzed as a continuous variable in all tests to maintain statistical power and prevent arbitrary grouping. The study was not intended to produce age-specific prevalence rates; instead, age was included as a covariate to control for potential confounding. This analytic approach consists of best practices for observational studies with wide age distributions. The final analytic sample comprised 909 participants, aged 18 to 93 years.

### 2.4. Measures and Instruments

*Sociodemographic and health behaviors*. Sociodemographic data (age, gender, marital status, educational attainment, employment status, annual household income) were collected using a standardized questionnaire adapted from the Philippine National Demographic and Health Survey (NDHS) [13]. Access-related variables included health insurance coverage and selected indicators of healthcare access. These variables were included to characterize the sample and to evaluate their contribution to pain severity in multivariable models. Health behaviors were self-reported (current smoking, alcohol use), and physical activity and sedentary behavior were captured as weekly exercise duration and daily sedentary time. Self-efficacy for managing health was measured using a single 10-point item (1 = not at all confident, 10 = very confident) for each of the following areas: blood pressure, cholesterol, nutrition, exercise, and weight. Key instruments, the exact items used, scoring procedures, and applicable notes are summarized in Table 1.

*Pain*. Pain severity was measured with the Numeric Rating Scale (NRS-11; 0 = no pain, 10 = worst imaginable), which demonstrated good to excellent test–retest reliability (Intraclass coefficient = 0.885) among those with chronic pain [14,15,16]. Chronic pain was defined a priori as pain present on most days for ≥3 months; respondents who screened positive for chronicity reported current pain intensity on the NRS. For descriptive purposes, NRS scores were categorized as 0 = none, 1–3 = very mild to mild, 4–6 = moderate, and 7–10 = extreme. Pain interference was measured using the Brief Pain Inventory interference (BPI) items, which were shown to have high internal consistency among those with chronic non-malignant pain (Cronbach’s alpha = 0.88 for interference subscale) [17]. The parent survey did not capture pain site(s) or clinician-attributed etiology; analyses, therefore, treat pain severity and interference as global constructs.

*Psychosocial, mood, and sleep measures*. Depression and anxiety symptoms were screened using the Hospital Anxiety and Depression Scale (HADS: HADS-A and HADS-D, each scored 0–21) [18]. The HADS demonstrated acceptable internal consistency (Cronbach’s alpha for HADS-A = 0.83 and HADS-D = 0.84) [19]. Sleep was evaluated using selected items from the Pittsburgh Sleep Quality Index (PSQI), including sleep onset latency, nocturnal awakenings, average nightly sleep duration, and subjective non-restorative sleep [20]. Health-related QoL was measured using the Short Form-12 (SF-12), which provides Physical Component Summary (PCS) and Mental Component Summary (MCS) scores standardized to a mean of 50 (Cronbach’s alpha for PCS = 0.85 and MCS = 0.84) [21].

### 2.5. Translation and Psychometric Considerations

Survey instruments were administered in English and/or Filipino, depending on what was appropriate as preferred by the participants. English language proficiency is high in the Philippines including rural regions. Where Filipino versions were available, previously translated and culturally adapted instruments were used. For instruments without formally validated Filipino versions at the time of data collection, translation procedures followed standard forward-translation and review processes to ensure conceptual equivalence. Because not all abbreviated or adapted items have comprehensive local psychometric data, we report internal reliability estimates for key scales when available and discuss measurement limitations in the limitations section.

### 2.6. Statistical Analysis

Descriptive statistics include participant characteristics, pain intensity, and psychosocial, sleep, and functional health metrics. Continuous data are presented as means with standard deviations, while categorical variables are reported as frequencies and percentages.

Bivariate relationships between pain severity and independent variables were examined using Pearson correlation coefficients for continuous variables and appropriate parametric tests for categorical variables. Results are displayed with 95% confidence intervals (CIs) when applicable.

Hierarchical linear regression models were constructed to evaluate the independent effects of psychological and clinical factors on pain severity. Model 1 integrated sociodemographic variables, such as age, sex, education, income, employment, and insurance status, to evaluate the variance exclusively attributable to demographic characteristics. Model 2 combined psychological and clinical factors, such as depressive symptoms, anxiety, sleep problems, and physical functioning, to determine their additional impact beyond demographic drivers. Changes in explained variance (Δ*R*^2^) were used to determine the additional explanatory value of psychosocial and clinical domains.

Model assumptions were evaluated before the analysis. Multicollinearity diagnostics indicated acceptable levels for all predictors (variance inflation factors < 2.5). Statistical significance was defined as a two-sided *p* < 0.05. All analyses were conducted using SPSS (version 27; IBM Corp., Armonk, NY, USA) [22].

### 2.7. Ethical Considerations

The I-HELP-FILIPINO study was approved by the University of California, Irvine Institutional Review Board (HS #2016-2761) and the University of the Philippines Manila Research Ethics Board (UPMREB #2016-496-01). All participants provided written informed consent before participation. The present study is a secondary analysis conducted under these approvals and within the scope of the original informed consent. No new data were collected for this analysis. All data were de-identified before analysis to protect participant confidentiality and privacy.

## 3. Results

### 3.1. Participant Characteristics

The descriptive analyses are based on 909 enrolled participants with complete data on pain intensity and core covariates (Table 2). The sample had a mean age of 53.8 years (SD 16.0); 63.1% of the participants were female, 63.5% were married, and 91.9% were uninsured. The annual household income was skewed, with 49.6% of respondents reporting an income of ≤PHP 50,000 per year (Table 2).

### 3.2. Pain Prevalence and Severity

Chronic pain was common: 83.8% reported pain on most days for ≥3 months (Table 3). By severity category, 16.2% (*n* = 147) were pain-free, 56.5% (*n* = 514) reported very mild to mild pain (NRS 1–3), 21.7% (*n* = 197) reported moderate pain (NRS 4–6), and 5.6% (*n* = 51) reported severe pain (NRS 7–10) (Table 3). Mean NRS pain score was 4.0 ± 1.9, and mean BPI score was 2.6 ± 1.3; itemized interference domains appear in Table 3. Psychosocial distress and sleep disturbances were prevalent in the sample, with mean scores for depression, anxiety, and sleep quality reflecting a heightened symptom load compared to population norms. Table 2 presents descriptive statistics for pain severity, psychosocial variables, sleep disturbances, and physical functioning.

### 3.3. Pain Severity and Correlations with Key Variables

Bivariate analyses (Table 4) showed that higher pain severity correlated most strongly with poorer physical QoL and with worse mood symptoms. SF-12 PCS had the largest bivariate association with pain (*r* = −0.718, 95% CI −0.748 to −0.685, *p* < 0.001). Higher pain was also associated with higher HADS-D and HADS-A scores and with several sleep problems (difficulty falling asleep, more nocturnal awakenings, non-restorative sleep, and shorter sleep duration). Lower weekly exercise time and lower confidence in exercising showed modest inverse associations with pain severity; education, income, smoking, and alcohol use were not meaningfully correlated with pain severity. Pairwise correlations indicated some overlap among PCS, mood, and sleep measures, but Spearman’s rho for skewed variables yielded similar effect sizes (Table 4).

Multivariable results from complete-case hierarchical regression are presented in Table 5 (model 2, *N* = 883).

Model 1 explained a minimal amount of variance (*R*^2^ = 0.029). Adding the prespecified clinical and psychosocial block substantially increases the explained variance: model 2 explained 55.9% (*R*^2^ = 0.559; adjusted *R*^2^ = 0.552; *F* (13,887) = 86.32, *p* < 0.001) (Table 5). In the fully adjusted complete-case model, lower SF-12 PCS was the strongest independent predictor of greater pain intensity. Difficulty falling asleep and higher depressive symptoms remained independently associated with higher pain; anxiety showed a smaller independent association. Female sex was associated with slightly lower pain scores. Age, income, average hours of sleep, nocturnal awakenings, non-restorative sleep, and SF-12 MCS did not independently predict pain in the adjusted model. Collinearity diagnostics indicated acceptable multicollinearity (VIFs < 2.5) and regression diagnostics supported model assumptions.

**Figure 2:**
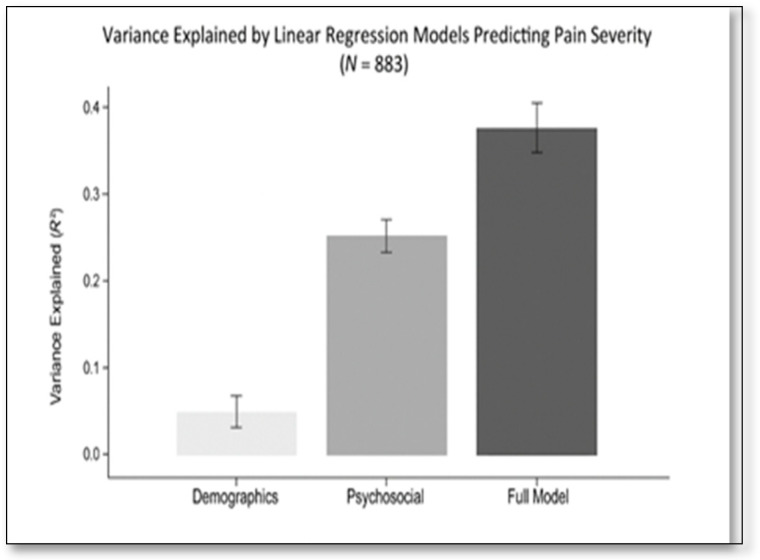
Variance explained by regression models predicting pain severity (*N* = 883). Model 1 = demographics only; model 2 (Full Model) = demographic, clinical, and psychosocial predictors. *R*^2^ values shown are from hierarchical linear regression predicting current pain severity (NRS); Model definitions are further described in Methods.

To address concerns about missing data, we performed multiple imputations (m = 20) as a sensitivity check. The pooled estimates from the imputed datasets yielded point estimates similar to those from the complete-case results and identical substantive conclusions. For transparency, the multivariable Table 5 and Figure 2 present the complete-case results (model 2, *N* = 883), and the captions note that the imputation sensitivity analyses were consistent with these results.

Figure 2 summarizes the incremental variance explained by the hierarchical modeling blocks (model 1: demographics only; psychosocial/clinical only; model 2: demographics and psychosocial/clinical predictors), visually emphasizing the substantially larger contribution of the psychosocial/clinical block to the explained variance (see Table 5 for Δ*R*^2^ and model details).

## 4. Discussion

This study investigated chronic pain and its biopsychosocial factors among 909 adults living in underserved rural Filipino communities. We observed a high rate of chronic pain and found that psychosocial, sleep, and functional health factors accounted for much more variation in pain severity than demographic factors. Reduced physical functioning, challenges in sleep initiation, and increased depressive symptoms emerged as the most consistent independent factors linked to higher pain severity. Anxiety showed a weaker independent connection, while female sex was associated with slightly lower pain scores in adjusted models. These findings support the biopsychosocial model of pain, highlighting that chronic pain intensity is primarily influenced by modifiable clinical and psychosocial factors rather than fixed demographic characteristics [1,23,24].

Study findings align with population-based studies from high- and middle-income countries, which show that sleep problems, depressive symptoms, and functional impairment are major correlates of chronic pain severity and disability [1,23,25]. Sleep disturbance has a bidirectional, though often asymmetric, relationship with pain, where poor sleep can lead to increased pain severity and interference over time [26,27]. Our findings extend this strong link between sleep problems and pain to rural Filipino populations, who are typically underrepresented in quantitative pain studies.

This study also found a particularly high link between functional health and pain severity. The strong link between SF-12 Physical Component Summary scores and pain is likely due to both the disabling effects of pain and partial construct overlap between global physical health indices and pain-related interference, as reported in other community-based studies using generic health status measures [1,23]. While age and gender are associated with pain outcomes in some large international surveys, their influence was limited in our models after accounting for psychosocial and clinical factors. These findings support the idea that clinical and psychosocial factors play a dominant role in determining chronic pain severity [4,24].

Comparing our findings with studies from rural Asia and Africa helps put them into context. These studies consistently show high rates of chronic pain that are linked to reduced physical functioning, poor sleep, and depression [25,28,29]. When comparing rural and urban populations, research suggests that ongoing pain and slow recovery in rural areas are often caused by structural barriers, limited access to nonpharmacologic treatments, and socioeconomic pressures rather than demographic factors [30]. Our findings support these observations and underscore the need to incorporate psychosocial and functional health considerations in developing pain management strategies tailored to rural Filipino populations.

### 4.1. Clinical and Assistive Practice Implications

The findings have significant implications for clinical and assistive practices in rural and resource-limited environments. A concise biopsychosocial screening, encompassing evaluations of physical functioning, sleep quality, and depressive symptoms, may help identify patients at risk of significant pain and functional disruption in primary care and community outreach settings [1,27]. In regions where specialized pain services are scarce, practical approaches such as task-sharing, telehealth-assisted behavioral therapies, and community-based exercise or rehabilitation programs may be effective methods for managing pain [24,30]. Interventions targeting sleep, mood, and functional ability could decrease pain-related impairment and warrant testing within rural Filipino communities [23,26].

### 4.2. Strengths and Limitations

This study boasts several strengths: A sizable sample from various rural barangays, the use of validated self-report tools appropriate for community environments, and a predefined biopsychosocial framework that considered the impact of psychosocial and clinical factors beyond basic demographic variables. However, some limitations need acknowledgment. The cross-sectional design limits causal inference and prevents determining the direction of relationships among sleep, mood, function, and pain. Convenience sampling and clinic-based recruitment restrict the generalizability of findings to similarly underserved communities. Dependence on self-report measures and specific instrument components may lead to measurement inaccuracies, and the lack of data on pain site or etiology necessitates treating pain as a general severity construct. Strong correlations between physical functioning and pain suggest potential construct overlap, even when multicollinearity diagnostics are acceptable.

### 4.3. Future Directions

Future research should focus on longitudinal studies to clarify the causal links between sleep, mood, functional health, and pain severity. Additionally, conducting pragmatic trials on low-intensity, scalable therapies in rural areas is essential. Research focused on implementation that emphasizes reach, cost-effectiveness, and equity—particularly via task-sharing and telehealth—is critical for developing sustainable pain management solutions for underserved populations [24,30].

## 5. Conclusions

This study reveals a significant prevalence of chronic pain among medically underserved rural Filipino people, indicating that psychosocial, sleep, and functional health factors—rather than demographic characteristics—primarily explain the diversity in pain severity. The findings quantify the additional impact of psychosocial and clinical factors beyond demographics, identifying measurable and modifiable targets, such as physical functioning, sleep disturbances, and depressive symptoms, that can be effectively screened and addressed in low-resource environments. These findings hold significant ramifications for healthcare provision and policy in the Philippines. Incorporating concise biopsychosocial screenings into primary care and community outreach, enhancing access to low-intensity behavioral and functional interventions, and coordinating with PhilHealth and local resources to facilitate nonpharmacologic care may alleviate the pain burden and improve daily functioning in rural communities. Overall, addressing sleep, mood, and functional health represents a practical, evidence-informed strategy for advancing equitable chronic pain care in underserved settings.

## Figures and Tables

**Table 1:** Instruments and key scoring.

Instrument	Construct Measured	Scoring/Cut points	Notes
Numeric Rating Scale (NRS-11)	Current Pain Severity	0–10 (0-none 1–3 very mild 4–6 moderate 7–10 extreme)	Chronic pain is defined as pain on most days ≥ 3 months
Brief Pain Inventory (BPI)	Pain interference	7 items are scored 0–10, and the mean is taken; higher scores = more pain interference	Used as a global functional impact measure
Hospital Anxiety and Depression Scale (HADS)	Anxiety and Depressive Symptoms: HADS-A (Anxiety) HADS-D (Depression)	Each subscale consists of 7 items (a total of 21 points) More than 8 in each subscale is suggestive of caseness	Screen only; not diagnostic
Pittsburgh Sleep Quality Index (PSQI)	Sleep problems: sleep onset latency; nocturnal awakenings; average nightly sleep duration; non-restorative sleep	Latency/awakenings (ordinal) Duration (continuous) Non-restorative sleep (ordinal)	Selected PSQI items were used instead of the full PSQI
Short Form-12 (SF-12)	Health-related quality of life: Physical Component Score (PCS) Mental Component Score (MCS)	Both are continuous variables and are standardized to a mean of 50	Abbreviated version of the SF-36
Single-item self-efficacy questions	Confidence in managing health behaviors	Five 10-point items 1 = not at all confident 10 = very confident	Items for BP, cholesterol, nutrition, exercise, and weight
Demographics and health behaviors questionnaire	Age, sex, marital status, education, income, insurance, smoking, alcohol, weekly exercise, daily sedentary time	Varied: continuous or categorical as described in Methods	Adapted from the Philippine NDHS

**Table 2:** Sociodemographic and Self-reported Data (*N* = 909).

Characteristic	*n* (%) or Mean ± SD
Age (years)	53.8 ± 16.0
Gender, female	574 (63.1%)
Married	577 (63.5%)
Education: ≤high school	665 (73.2%)
Annual income ≤ PHP 50,000 Uninsured	444 (49.6%) 835 (91.9%)
Mean NRS pain score	4.0 ± 1.9
Mean BPI interference	2.6 ± 1.3
HADS-D	4.9 ± 4.1
HADS-A	6.6 ± 3.7
SF-12 PCS	46.9 ± 7.9
SF-12 MCS	49.4 ± 7.3
Weekly exercise time (hours)	2.6 ± 1.0

Notes: Values are presented as *n* (%) unless otherwise indicated. Continuous variables are presented as mean (standard deviation). Chronic pain was defined as pain persisting for ≥3 months. NRS = Numeric Rating Scale (0–10). BPI = Brief Pain Inventory; higher scores indicate greater pain severity. HADS = Hospital Anxiety and Depression Scale; higher scores indicate greater symptom severity. SF-12 PCS = 12-Item Short Form Health Survey Physical Component Summary; lower scores indicate poorer physical functioning. Percentages may not total 100% due to rounding or missing data.

**Table 3:** Chronic pain prevalence and interference (*N* = 909).

	*n*	%
Pain Category		
None	147	16.2
Very mild to mild	514	56.5
Moderate	197	21.7
Extreme	51	5.6
Interference with Work		
Not at all	147	16.2
Slightly	514	56.5
Moderately	197	21.7
Quite a bit	37	4.1
Extremely	14	1.5

Notes: Values are presented as *n* (%) unless otherwise indicated. Chronic pain was defined as pain persisting for ≥3 months. BPI = Brief Pain Inventory. BPI interference items assess the extent to which pain interferes with daily activities (e.g., general activity, mood, walking ability, work, relationships, sleep, and enjoyment of life) on a 0–10 scale, with higher scores indicating greater interference. Percentages may not total 100% due to rounding.

**Table 4:** Correlations Between Pain and Key Variables.

Variable	*r*	95% CI	*p*	*N*
Age	0.169	0.106 to 0.233	<0.001	901
Sedentary time	0.150	0.086 to 0.214	<0.001	901
Exercise time	−0.300	−0.359 to −0.239	<0.001	901
Confidence in exercising	−0.370	−0.427 to −0.312	<0.001	901
Confidence in nutrition	−0.200	−0.261 to −0.137	<0.001	901
Depression (HADS-D)	0.360	0.301 to 0.415	<0.001	901
Anxiety (HADS-A)	0.390	0.333 to 0.443	<0.001	901
Difficulty falling asleep	0.280	0.219 to 0.340	<0.001	901
Night awakenings	0.247	0.188 to 0.305	<0.001	901
Non-restorative sleep	0.341	0.281 to 0.397	<0.001	901
Average hours of sleep	−0.224	−0.291 to −0.168	<0.001	901
Quality of life—physical (SF-12 PCS)	−0.718	−0.751 to −0.688	<0.001	901
Quality of life—mental (SF-12 MCS)	−0.303	−0.349 to −0.228	<0.001	901

Notes: Pearson correlation coefficients (*r*) are shown. *N* = 901. Ninety-five percent confidence intervals (95% CIs) were calculated using Fisher’s *z* transformation and back transformed to the *r* scale. All *p* values are two-tailed. HADS = Hospital Anxiety and Depression Scale; higher scores indicate greater symptom severity. PCS = Physical Component Summary score of the SF-12; higher scores indicate better physical functioning. Strong correlations between pain severity and physical functioning (e.g., PCS with pain, *r* = −0.718) reflect substantial association between constructs; the potential for construct overlap is considered in the Discussion and Limitations. Multicollinearity diagnostics for subsequent regression analyses indicated acceptable levels (variance inflation factors < 2.5).

**Table 5:** Model 2: Multivariable linear hierarchical regression predicting pain.

Predictor	*B*	SE	95% CI	*β*	*p*
Physical quality of life	−0.058	0.003	−0.064 to −0.052	−0.612	<0.001
Difficulty falling asleep	0.080	0.023	0.035 to 0.125	0.108	<0.001
HADS depression score	0.051	0.014	0.024 to 0.078	0.092	<0.001
HADS anxiety score	0.015	0.006	0.003 to 0.027	0.083	0.007
Gender (female = 1)	−0.086	0.037	−0.159 to −0.013	−0.055	0.020

Notes: Unstandardized regression coefficients (*B*) with 95% confidence intervals (CIs) are shown. Model 2 includes sociodemographic variables and psychosocial and clinical variables. Δ*R*^2^ represents the change in explained variance between models. HADS = Hospital Anxiety and Depression Scale; higher scores indicate greater symptom severity. All *p* values are two-tailed. Variance inflation factors were <2.5 for all predictors, indicating acceptable multicollinearity.

## Data Availability

The raw data supporting the conclusions of this article will be made available by the authors on request due to privacy reasons.

## Abbreviations

The following abbreviations are used in this manuscript:

U.S.	United States
E.g.	For example
STROBE	Strengthening the Reporting of Observational Studies in Epidemiology
NDHS	Philippine National Demographic and Health Survey
NRS-11	Numeric Rating Scale-11
BPI	Brief Pain Inventory
HADS	Hospital Anxiety and Depression Scale
HADS-A	Hospital Anxiety and Depression Scale, Anxiety subscale
HADS-D	Hospital Anxiety and Depression Scale, Depression subscale
PSQI	Pittsburgh Sleep Quality Index
SF-12	Short Form-12
SF-36	Short Form-36
PCS	Physical Component Summary
MCS	Mental Component Summary
BP	Blood Pressure
QoL	Quality of Life
CI	Confidence Interval
Δ*R*^2^	Change in explained variance
*P*	Probability value
*SPSS*	Statistical Package for the Social Sciences
IBM	International Business Machines
NY	New York
USA	United States of America
SD	Standard Deviation
PHP	Philippine Peso
*N*	Total population size
*n*	Sample size
*R*	Pearson correlation coefficient
*B*	Unstandardized regression coefficient
SE	Standard Error
*β*	Standardized Regression Coefficient
*R*^2^	Coefficient of determination of a multiple regression
*R*	Multiple correlation coefficient
VIF	Variance Inflation Factors
